# Draft genome assembly of *Passalora sequoiae* a needle blight pathogen on Leyland cypress

**DOI:** 10.1186/s13104-020-05328-3

**Published:** 2020-11-04

**Authors:** Warren E. Copes, Jorge Ibarra Caballero, Ebrahiem Babiker, Jane E. Stewart, Valerie A. Orner, Alan S. Windham, Renee S. Arias

**Affiliations:** 1 USDA ARS Thad Cochran Southern Horticultural Laboratory, Poplarville, MS 39470 USA; 2 Dept. of Agricultural Biology, Colorado State University, Fort Collins, CO 80523 USA; 3 USDA ARS National Peanut Research Laboratory, Dawson, GA 39842 USA; 4 Soil, Plant and Pest Center, University of Tennessee, Nashville, TN 37211 USA

**Keywords:** *Cupressocyparis leylandii*, Genome annotation, Illumina, Leyland cypress, Needle blight, PacBio

## Abstract

**Objective:**

*Passalora sequoiae* (family Mycosphaerellaceae) causes a twig blight on Leyland cypress that requires numerous fungicide applications annually to minimize economic losses for ornamental plant nursery and Christmas tree producers. The objective was to generate a high-quality draft assembly of the genome of *P. sequoiae* as a resource for primer development to investigate genotype diversity.

**Data description:**

We report here the genome sequence of *P. sequoiae* 9LC2 that was isolated from Leyland cypress ‘Leighton Green’ in 2017 in southern Mississippi, USA. The draft genome was obtained using Pacific Biosciences (PacBio) SMRT and Illumina HiSeq 2500 sequencing. Illumina reads were mapped to PacBio assembled contigs to determine base call consistency. Based on a total of 44 contigs with 722 kilobase (kb) average length (range 9.4 kb to 3.4 Mb), the whole genome size was estimated at 31,768,716 bp. Mapping of Illumina reads to PacBio contigs resulted in a 1000 × coverage and were used to confirm accuracy of the consensus sequences.

## Objective

**Fig. 1:**
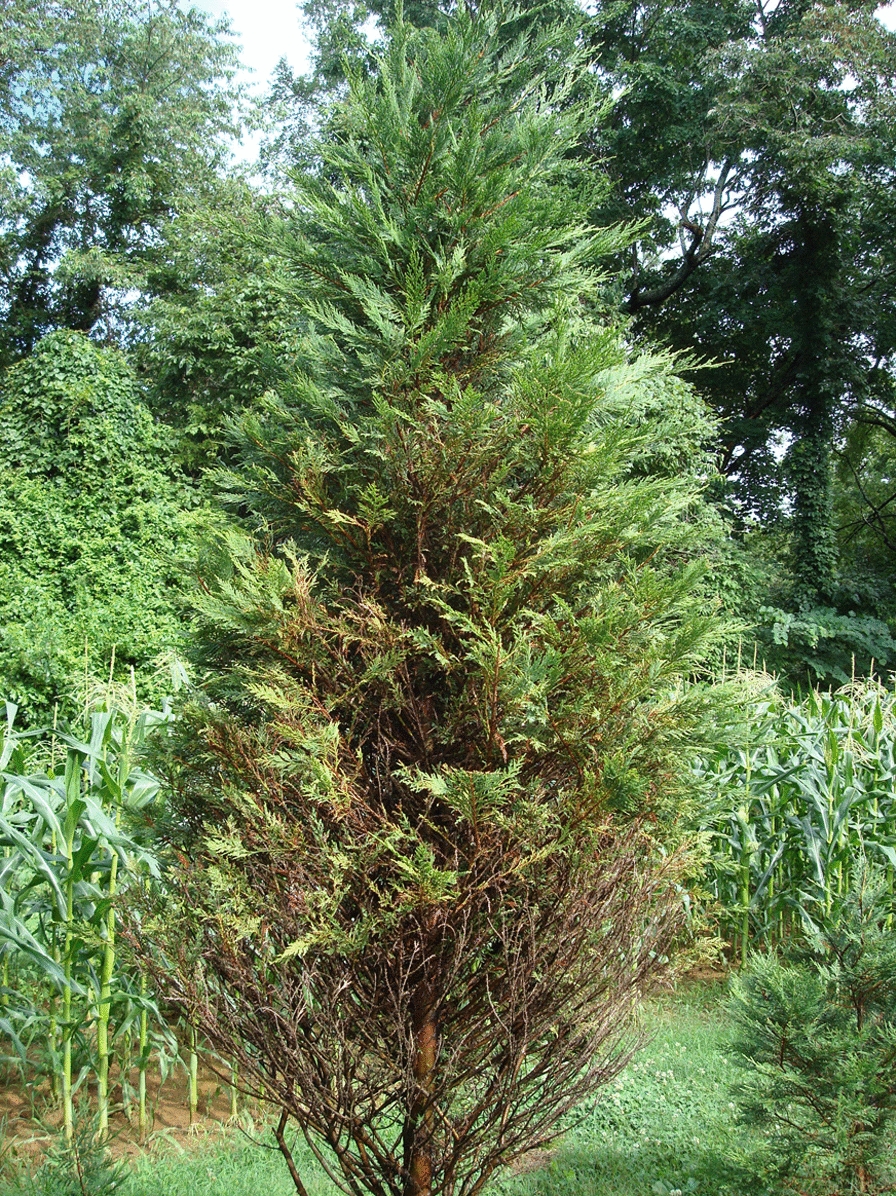
Leyland cypress tree showing Passalora twig blight symptoms

*Passalora sequoiae* (Ellis & Everh.) Y.L. Guo & W.H. Hsieh (syn. *Cercosporidium sequoiae* (Ellis and Everh.) Baker and Partridge) is a fungus that causes needle blight on genera in the *Cupressaceae*, mainly *Leyland cypress* (x *Cupressocyparis leylandii*) [1, 2]. Disease symptoms of brown to gray needles appear during the spring and progressively appear throughout the tree canopy to result in unmarketable trees (Fig. 1). Annual fungicide application and crop loss inflict significant costs on the ornamental tree and Christmas tree industries [3–5].

The objective of this work was to sequence the whole genome of *P. sequoiae* using PacBio and Illumina to assemble contigs. A lack of genetic information for this fungus prevents utilization of genetic tools to determine genetic diversity of isolates, potential differences in virulence, and ultimately the development of control practices. Currently, only three entries are listed for *Passalora* spp. in GenBank (NCBI), corresponding to the 18S rDNA gene of this fungus, a total of 5476 base pairs (bp).

**Fig. 2:**
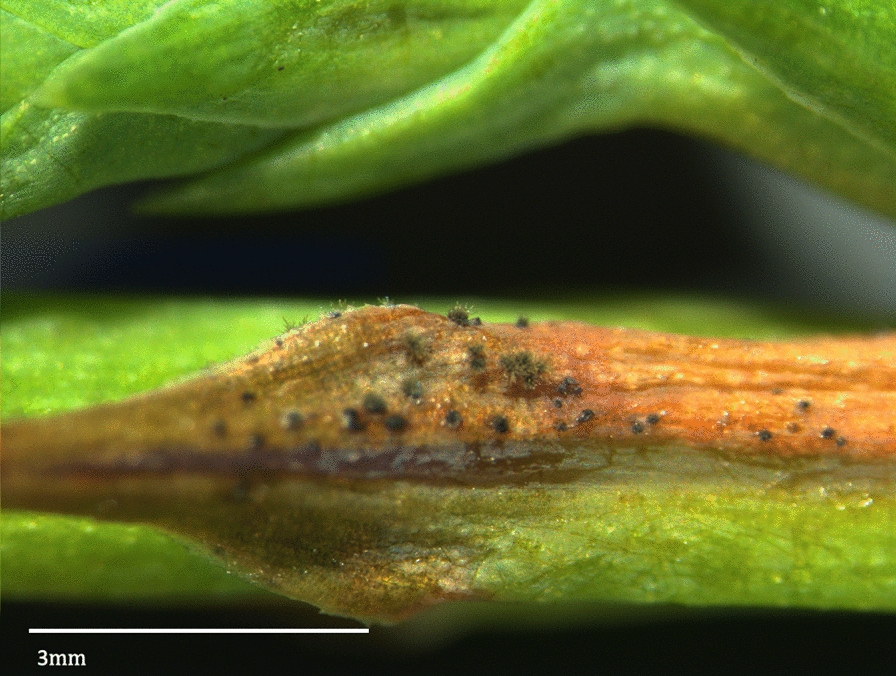
Infected Leyland cypress leaf with sporulating conidioma of *Passalora sequoiae*

**Fig. 3:**
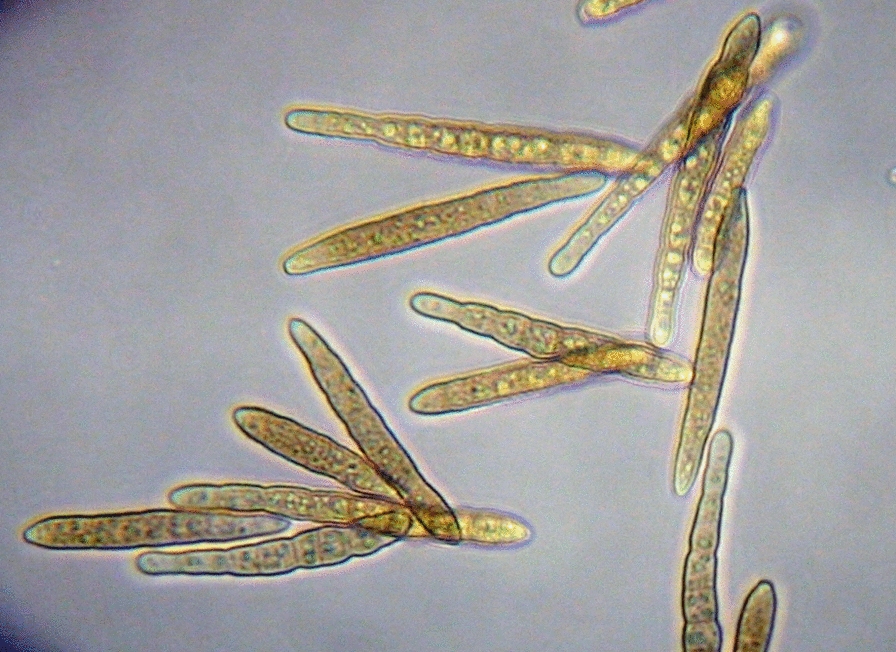
Conidia of *Passalora sequoiae*

A problem in sampling *P. sequoiae* populations is that numerous dematiaceous hyphomycetes with morphologically similar conidia and conidioma are found in many regions (Figs. 2 and 3). Proper identification of these organisms is further complicated by the numerous name revisions over the last two decades [1, 6–12]. A further constraining factor is that only a small number of dematiaceous hyphomycetes have been included in genetic phylogenies using DNA loci, mRNA and proteins [7, 10–20]. *Mycosphaerellaceae* was recently narrowed to 120 genera based on phylogenetic data [12].

## Data description

A single spore isolate of *P. sequoiae* 9LC2 was recovered from a Christmas tree near Hattiesburg, MS, USA. DNA was extracted [21] and sheared to approximately 20 kb fragments. SMRTbell library was prepared, then sequenced on a PacBio Sequel sequencer at USDA-ARS, Stoneville, MS, USA. Bam files were processed using Finishing Module 20.0 of CLC_Bio Workbench v.12 (Qiagen LLC, Hilden, Germany). A total of 519,499 subreads with 6,612,712,889 nucleotides (nt) total, average length 14,247 nt, N50 21,720, were generated. Subreads were corrected and de novo assembled. The initial 19 contigs were manually split when necessary, rendering 44 contigs of 722,016 nt average and 44 x coverage. A total of 244,368,646 reads with an average length of 148 nt after trimming were obtained from Illumina sequencing. These reads were mapped to the PacBio assembled contigs resulting in 1011 x average coverage. A small percentage of gaps, 2–4 nt in length, approximately 2–3 gaps every 150,000 nt were observed using Illumina reads on the PacBio assembly, and they corresponded to microsatellites; thus, in all cases, the PacBio assembly was chosen (Table 1).

**Table 1:** Overview of data files/data sets

Label	Name of data file/data set	File types (file extension)	Data repository and identifier (DOI or accession number)
Data file 1	ALL_CONTIGS_Passalora_sequoiae_RenamedDec12_2019.fsa	FASTA (.fsa)	GenBank Accession: https://identifiers.org/ncbi/insdc:WSQC01000000 [33]
Data set 1	Fig. 1 LC blight symptoms	.jpg	DOI: https://www.doi.org/10.15482/USDA.ADC/1518905 [34]
Data set 1	Fig. 2 Passalora sporulation	.png	DOI: https://www.doi.org/10.15482/USDA.ADC/1518905 [34]
Data set 1	Fig. 3 Passalora conidia	.png	DOI: https://www.doi.org/10.15482/USDA.ADC/1518905 [34]
Data set 1	Fig. 4 Passalora 9LC2 phylogeny	.pdf	DOI: https://www.doi.org/10.15482/USDA.ADC/1518905 [34]
Data set 1	Methodology	WORD (.docx)	DOI: https://www.doi.org/10.15482/USDA.ADC/1518905 [34]

**Fig. 4:**
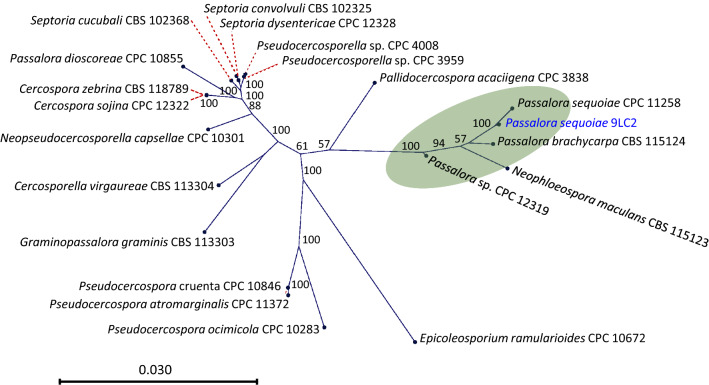
Phylogeny of *Passalora sequoiae* 9LC2 and closely related species based on Neighbor-Joining analysis of 5465 nt of 18S ribosomal RNA (rRNA) gene, Internal transcribed spacer (ITS) 1, 5.8S rRNA gene, ITS2 and 28S ribosomal RNA gene partial sequence. Bootstrap of 100 resampling are shown at the nodes; scale is nucleotide substitution rate

Basic Local Alignment Search Tool (BLAST) [22] of a 9360 nt contig containing the 18S rDNA gene and internal transcribed spacers of *P. sequoiae* isolate 9LC2 showed a 99.65% identity with the 5476 nt NCBI entry *Passalora sequoiae* GU214667.1 [10]. The 5476 bp region of 9LC2 was used to retrieve 20 closely related sequences with 100% coverage. A Neighbor Joining [23] phylogenetic radial tree was constructed [24] using CLC Genomics Workbench 20.0 (Fig. 4), using NCBI accessions: GU214655.1; GU214656.1; GU214658.1; GU214661.1; GU214662.1; GU214664.1; GU214665.1; GU214666.1; GU214667.1; GU214668.1; GU214670.1; GU214671.1; GU214673.1; GU214678.1; GU214684.1; GU214686.1; GU214688.1; GU214697.1; GU214698.1; GU214699.1. *Passalora sequoiae* 9LC2 showed 99.7% identity to *P. sequoiae* CPC 11258, and 99.2 identity to *P. brachycarpa* CBS 115124. Though the taxonomy of *Passalora* is still being debated [12], *P. sequoiae* 9LC2 grouped with previously reported *Passalora* spp.

Structural annotation of the genome assembly was determined using MAKER v.2.31.8 [25]. The MAKER pipeline included programs 1) RepeatMasker v.4.0.6 [26] to mask interspersed repeats and low complexity DNA sequences; 2) three gene predictors: GeneMark-ES [27]; SNAP [28], trained with Sordariomycetidae proteins from the Uniprot database; and Augustus [29]; and 3) tRNAscan [30] to identify tRNA genes in the genomic sequence. The total number of genes identified by Maker was 10,657. Of those, 10,576 genes were predicted to have proteins ≥ 50 amino acids. Maker also identified 81 tRNA and 3.42% of the genome corresponded to short repetitive sequences.

DbCAN2 [31] identified 331 predicted proteins that had signatures as carbohydrate active enzymes (CAZymes). Of those 52, 9, 186, 3, 79 and 9 corresponded to auxiliary activity enzymes, carbohydrate esterases, glycoside hydrolases, polysaccharide lyases, glycosyl transferases and carbohydrate binding modules, respectively. Thirty-four proteins had blast hits to the phi-database [32].

This whole-genome project has been deposited in DDBJ/ENA/GenBank under the accession number WSQC00000000 [33]. The version described in this paper is the first version, WSQC01000000.

## Limitations

The genome sequence of a single isolate of *P. sequoiae* is being reported; thus, sequences of additional isolates would be needed to perform comparative genomics. Mapping of the Illumina sequences to PacBio contigs resulted in small gaps of low frequency; therefore, no serious limitation of data quality was evident. Reconstruction of whole chromosomes showing predicted genes and their annotation would provide characterization of the structural and functional levels.

## Data Availability

This Whole Genome Shotgun project has been deposited at DDBJ/ENA/GenBank [33]. The version described in this paper is version https://identifiers.org/ncbi/insdc:WSQC01000000. Given size limitations for uploading, raw data are available from Renee.Arias@usda.gov upon reasonable request. Due to the extremely slow growth and nutritional requirements of this organism, the type strain has been stored at USDA-ARS Thad Cochran Southern Horticultural Laboratory, Poplarville, MS. The dataset of figures and the full methodology is available in the Ag Data Commons repository maintained by the United States Department of Agriculture [34].

## Abbreviations

NCBI: National Center for Biotechnology/Information
PacBio: Pacific Biosciences
BLAST: Basic Local Alignment Search Tool
